# A Reply to Comment on Kluckow M. Barriers to the Implementation of Newborn Pulse Oximetry Screening: A Different Perspective. *Int. J. Neonatal Screen.* 2018, *4*(1), 4

**DOI:** 10.3390/ijns4020014

**Published:** 2018-04-17

**Authors:** Martin Kluckow

**Affiliations:** Department of Neonatal Medicine, Royal North Shore Hospital and University of Sydney, Sydney, NSW 2065, Australia; martin.kluckow@sydney.edu.au; Tel.: +61-2-9463-2180

The commentary provided by Gentles et al. argues for the implementation of a universal pulse oximetry screening program, and I agree that, if it is possible, this is the optimum way to introduce this important health care measure for all of the reasons set out by the authors. However, there are significant impediments to achieving this in many countries, including in the author’s own country of New Zealand, where I understand that several large obstetric hospitals still do not have a full pulse oximetry screening program, pending an attempt at countrywide implementation. One could argue that this is decreasing access to this healthcare intervention, whilst attempting to provide a 100% solution for the whole population. A noble aim, but practically challenging—as has been shown already in the United Kingdom (UK), where, despite one of the world authorities on pulse oximetry screening leading the way towards universal pulse oximetry screening there, it has still not been achieved more than six years after the publication of seminal papers [1]. In fact, most UK hospitals seem to have been adopting the hospital-led screening discussed in my article, with only 19% of hospitals choosing to wait for the National recommendation—if it is achieved [1,2]. I acknowledge that hospital-led screening is a less robust system, but sometimes the gold standard solution is not practical. The issues raised as to time and cost of consumables have been easily overcome in the majority of centres in Australia, at all tiers of health care, as pulse oximetry measurement has become more available and increasingly utilised in postnatal wards for other purposes, apart from formal screening. Audit of the program has been achieved by incorporating screening information into local and national perinatal data collections. The majority of babies born in Australia now undergo pulse oximetry screening. I wish the authors well in achieving their aim of universal pulse oximetry screening in New Zealand.
